# BMC Plant Biology reviewer acknowledgement 2014

**DOI:** 10.1186/s12870-015-0418-4

**Published:** 2015-03-07

**Authors:** Catherine J Potenski

**Affiliations:** BioMed Central, 233 Spring Street, New York, NY 10013-1578 USA

**Tabare Abadie**

USA

**Steffen Abel**

Germany

**Amanda Able**

Australia

**Gaber Abogadallah**

Egypt

**Maricelis Acevedo**

USA

**Anne-Françoise Adam-Blondon**

France

**Keith Adams**

Canada

**Oussama Ahrazem**

Spain

**Riccardo Aiese Cigliano**

Spain

**Karen Aitken**

Australia

**Takashi Akagi**

Japan

**Eduard Akhunov**

USA

**Magdy Alabady**

USA

**Salim Al-Babili**

Saudi Arabia

**Maria Albani**

Germany

**Nick Albert**

New Zealand

**Ruben Alcazar**

Spain

**Juan De Dios Alché**

Spain

**Ross Alexander**

UK

**Pablo Aleza**

Spain

**Francisco Alfocea**

Spain

**Andrew Allan**

New Zealand

**Doug Allen**

USA

**Jose M Alvarez**

Spain

**Sara Amancio**

Portugal

**Erik Andreasson**

Sweden

**Fernando Andres Lalaguna**

Germany

**Aldwin Anterola**

USA

**Carla Antonio**

Portugal

**Baltazar Antonio**

Japan

**Michael N Antoniou**

UK

**Pedro M Aparicio-Tejo**

Spain

**Rudi Appels**

Australia

**Miguel Aranda**

Spain

**Gen-Ichiro Arimura**

Japan

**Grace Armijo**

Chile

**Vincent Arondel**

France

**Michael Axtell**

USA

**Belay Ayele**

Canada

**Ricardo Azevedo**

Brazil

**Andreas Bachmair**

Austria

**Sacha Baginsky**

Germany

**Agnieszka Bagniewska-Zadworna**

Poland

**Yuling Bai**

Netherlands

**Donovan Bailey**

USA

**Fabienne Baillieul**

France

**Barbara Baker**

USA

**Salma Balazadeh**

Germany

**Alma Balestrazzi**

Italy

**Peter Balint-Kurti**

USA

**Tomohiro Ban**

Japan

**Pradeepa Bandaranayake**

Sri Lanka

**Zsófia Bánfalvi**

Hungary

**Abdelali Barakat**

USA

**Elena Baraldi**

Italy

**William Barbazuk**

USA

**Todd Barkman**

USA

**Yves Barriere**

France

**Francisco Barro**

Spain

**Daniele Bassi**

Italy

**Soumalee Basu**

India

**Giorgia Batelli**

Italy

**Sébastien Baud**

France

**Dirk Becker**

Germany

**Eric Beers**

USA

**Angjelina Belaj**

Spain

**Catherine Bellini**

Sweden

**Francois Belzile**

Canada

**Abdelhafid Bendahmane**

France

**Gary Bending**

UK

**Vagner Benedito**

USA

**Moussa Benhamed**

France

**Eva Benkova**

Austria

**Jeffrey Bennetzen**

USA

**Leonie Bentsink**

Netherlands

**Mark Bernards**

Canada

**Frank Bernhard**

Germany

**Alexandre Berr**

France

**James Berry**

USA

**Sébastien Besseau**

France

**K.E. Bett**

Canada

**Michael Bevan**

UK

**Brad Binder**

USA

**Benjamin Blonder**

USA

**Christine Boettcher**

Australia

**Martin Bohn**

USA

**Torsten Bohn**

Luxembourg

**Cordelia Bolle**

Germany

**Victoria Bonnecarrere**

Uruguay

**Guusje Bonnema**

Netherlands

**Ljudmilla Borisjuk**

Germany

**Maria Borisova-Mubarakshina**

Russian Federation

**Paul Boss**

Australia

**Rebecca Boston**

USA

**Javier Botto**

Argentina

**Alessandro Botton**

Italy

**Marc Boutry**

Belgium

**Peter Bozhkov**

Sweden

**Peter Bradbury**

USA

**Andrea Braeutigam**

Germany

**Gintaras Brazauskas**

Lithuania

**Phil Bregitzer**

USA

**Dev Britto**

Canada

**Mikael Brosche**

Finland

**Cory Brouwer**

USA

**Robert Brueggeman**

USA

**Qingyun Bu**

China

**Thomas Buckhout**

Germany

**Mariano Bulos**

Argentina

**Nicola Busatto**

Italy

**Daniel Bush**

USA

**John Bussell**

Australia

**Patrick Byrne**

USA

**Francisco Cabrera-Chavez**

Mexico

**Tao Cai**

China

**Yingfan Cai**

China

**Myriam Calonje**

Spain

**Benjamin Campbell**

USA

**Michael Campbell**

USA

**Alexandre Campos**

Portugal

**Francisco R Canton**

Spain

**Dario Cantu**

USA

**Juan Carbonell**

Spain

**Pablo Carbonell-Bejerano**

Spain

**Maura Cardarelli**

Italy

**John Carman**

USA

**Sebastien Carpentier**

Belgium

**Joyce Cartagena**

Japan

**Clay Carter**

USA

**Carla Caruso**

Italy

**Luiz Carvalho**

Brazil

**Helena Carvalho**

Portugal

**Jorge Casal**

Argentina

**Simone Castellarin**

Canada

**David Chagne**

New Zealand

**Zhulong Chan**

China

**Yee-Yung Charng**

Taiwan

**Carolina Chavarro**

USA

**Kun-Song Chen**

China

**Shuangchen Chen**

China

**Dong-Hong Chen**

China

**Fadi Chen**

China

**Caiyan Chen**

China

**Fan Chen**

China

**Feng Chen**

USA

**Jinfeng Chen**

USA

**Michelle Y.Q. Chen**

China

**Yinglong Chen**

China

**Zhixiang Chen**

USA

**Riyan Cheng**

USA

**Ravindra Chibbar**

Canada

**Kevin Childs**

USA

**Maurizio Chiurazzi**

Italy

**Hyung-Taeg Cho**

Korea, South

**Giltsu Choi**

Korea, South

**Frederic Choulet**

France

**Wah Soon Chow**

Australia

**David Christopher**

USA

**Byung Yeoup Chung**

Korea, South

**Marta Cifuentes**

Spain

**Stephan Clemens**

Germany

**Sylvie Cloutier**

Canada

**Jeremy Coate**

USA

**Joseph Colasanti**

Canada

**David Collings**

New Zealand

**Thomas Colquhoun**

USA

**Eleonora Cominelli**

Italy

**Peter Constabel**

Canada

**Lucio Conti**

Italy

**Bret Cooper**

USA

**Sylvain Cordelier**

France

**Amandine Cornille**

Sweden

**Fabrizio Costa**

Italy

**Grant Cramer**

USA

**Martin Crespi**

France

**Xiangqin Cui**

USA

**Shaun Curtin**

USA

**Abdelfattah Dababat**

Turkey

**Hongyi Dai**

China

**Xinbin Dai**

USA

**Zhanwu Dai**

France

**Jonas Danielson**

Sweden

**Zsuzsanna Darula**

Hungary

**Sampa Das**

India

**Yasuhiro Date**

Japan

**Karine David**

New Zealand

**Christopher Davies**

Australia

**Kevin Davies**

New Zealand

**Stefan De Folter**

Mexico

**Paolo De Franceschi**

Italy

**Luit J. De Kok**

Netherlands

**Ruud De Maagd**

Netherlands

**Nico De Storme**

Belgium

**David De Vleesschauwer**

Belgium

**Sacco De Vries**

Netherlands

**Seth Debolt**

USA

**Nicolas Delhomme**

Sweden

**Costas Delis**

Greece

**Serge Delrot**

France

**Laurent Deluc**

USA

**Xiuxin Deng**

China

**Emanuel Devers**

Switzerland

**Paul Devlin**

UK

**Michael K Deyholos**

Canada

**Christelle Deytieux**

Belleau Reunion

**Sandrine Dhondt-Cordelier**

France

**Christophe D'Hulst**

France

**Gabriele Di Gaspero**

Italy

**Daniel Dias**

Australia

**Isabel Diaz**

Spain

**Jose Diaz**

Spain

**Scott Diehn**

USA

**Stephen Difazio**

USA

**Yi Ding**

China

**Assaf Distelfeld**

Israel

**Anna Dobritsa**

USA

**Agnes Doligez**

France

**Kara Dolinski**

USA

**James Dombrowski**

USA

**Lloyd Donaldson**

New Zealand

**Aiwu Dong**

China

**Zhenying Dong**

China

**Daolong Dou**

China

**Renato D'Ovidio**

Italy

**Max Dow**

Ireland

**Kurt Drickamer**

UK

**Hajk-Georg Drost**

Germany

**Arnaud Duhoux**

France

**Marcello Duranti**

Japan

**Melvin Duvall**

USA

**Charles Dwamena**

New Zealand

**Alan Eggenberger**

USA

**Malin Elfstrand**

Sweden

**Paula Elomaa**

Finland

**Francesco Emanuelli**

Italy

**Eugenia Enfissi**

UK

**Jurgen Engelberth**

USA

**Cawas Engineer**

USA

**Carmen Espinoza**

Chile

**Yoram Eyal**

Israel

**Muhammad Faheem**

Pakistan

**Osvaldo Failla**

Italy

**Aaron Fait**

Israel

**Chengming Fan**

China

**Liumin Fan**

China

**David Fang**

USA

**Eva Farre**

USA

**Jacqueline Farrell**

USA

**Attila Fehér**

Hungary

**Alejandro Ferrando**

Spain

**Alison Ferrie**

Canada

**Jan Fíla**

Czech Republic

**Sophie Filleur**

France

**Richard Finkers**

Netherlands

**Matthias Fladung**

Germany

**Andrew Flavell**

UK

**Jozsef Fodor**

Hungary

**Daniel Fonceka**

France

**Christopher Ford**

Australia

**Britta Forster**

Australia

**John Forster**

Australia

**Christine Foyer**

UK

**Michael Francki**

Australia

**Jerome Franckowiak**

USA

**Wolfgang Frank**

Germany

**Noni Franklin-Tong**

UK

**Susanne Franssen**

Germany

**Elisabetta Frascaroli**

Italy

**Margrit Frentzen**

Germany

**Florian Frugier**

France

**Julia Frugoli**

USA

**Daolin Fu**

China

**Feng-Ling Fu**

China

**Binying Fu**

China

**Yong-Fu Fu**

China

**Karen Fugate**

USA

**Hiroaki Fujii**

Finland

**Yasunari Fujita**

Japan

**Hiroyuki Fukuoka**

Japan

**Beata Gabrys**

Poland

**Agata Gadaleta**

Italy

**Giovanni Gadda**

USA

**Malgorzata Gaj**

Poland

**Carlos Galeano**

Belgium

**Gabor Galiba**

Hungary

**Ivan Galis**

Japan

**Giulio Galla**

Italy

**Vanessa Galli**

Brazil

**Susheng Gan**

USA

**Dongying Gao**

USA

**Junping Gao**

China

**Liangliang Gao**

USA

**Nigel Gapper**

USA

**Rohini Garg**

India

**Nicole Gaude**

Germany

**Danny Geelen**

Belgium

**Agustina Gentile**

Brazil

**Bernard Genty**

France

**Godelieve Gheysen**

Belgium

**Luca Gianfranceschi**

Italy

**Antonia Gibalova**

Czech Republic

**Markus Gierth**

Germany

**Javier Gil-Humanes**

USA

**Upinder Gill**

USA

**Lara Giongo**

Italy

**Giovanni Giuliano**

Italy

**Jeff Glaubitz**

USA

**Fred Gmitter**

USA

**Barry Goldfarb**

USA

**Vassiliy Goltsev**

Bulgaria

**John Golz**

Australia

**Nikolay P. Goncharov**

Russian Federation

**Daniel H. Gonzalez**

Argentina

**Carmen González**

Spain

**Luis González-Candelas**

Spain

**William Gordon-Kamm**

USA

**Francoise Gosti**

France

**Lydia Gramzow**

Germany

**Silvana Grandillo**

Italy

**Antonio Granell**

Spain

**David Grant**

USA

**Eric Grenier**

France

**Peter Gresshoff**

Australia

**Åsa Grimberg**

Sweden

**Andrew Groover**

USA

**Erich Grotewold**

USA

**Corrinne Grover**

USA

**Nicole Gruenheit**

UK

**Yong Qiang Gu**

USA

**Paul Gugger**

USA

**Sabine Guillaumie**

France

**Raghavendra Gunnaiah**

India

**Yan Guo**

China

**Hui Guo**

USA

**Om Gupta**

India

**Pushpendra Gupta**

India

**Juan Gutierrez-Gonzalez**

USA

**Jose Gutierrez-Marcos**

UK

**Carlos Guzman**

Spain

**Niclas Gyllenstrand**

Sweden

**János Györgyey**

Hungary

**Jeff Habben**

USA

**Charles Hachez**

Belgium

**Bernd Hackauf**

Germany

**Uwe Hacke**

Canada

**Ken Haga**

Japan

**Hely Häggman**

Finland

**Candace Haigler**

USA

**Ian Hallett**

New Zealand

**Noureddine Hamamouch**

USA

**Bjoern Hamberger**

Denmark

**Mei Han**

Canada

**Oksoo Han**

Korea, South

**Yuepeng Han**

China

**Yuko Hanba**

Japan

**Robert Hancock**

UK

**Marc Hanikenne**

Belgium

**Viola Hanke**

Germany

**L. Curtis Hannah**

USA

**Abdelali Hannoufa**

Canada

**Maureen Hanson**

USA

**Yoshie Hanzawa**

USA

**Phillip Harries**

USA

**Neil Harris**

Canada

**Philip Harris**

New Zealand

**Caroline Hartmann**

France

**Marie-Theres Hauser**

Austria

**Simon Hawkins**

France

**Matthew Haworth**

Italy

**Samuel Hazen**

USA

**Guangyuan He**

China

**Ping He**

USA

**Yuehui He**

China

**Yuke He**

China

**Zhonghu He**

China

**Reinhard Hehl**

Germany

**Alain Hehn**

France

**Martin Heil**

Germany

**Jan Hejatko**

Czech Republic

**Roger Hellens**

Australia

**Kian Hematy**

France

**Tracie Hennen-Bierwagen**

USA

**Jacek Hennig**

Poland

**Luis E. Hernandez**

Spain

**Georgina Hernández**

Mexico

**Sigrid Heuer**

Australia

**Tarek Hewezi**

USA

**Julian Hibberd**

UK

**Karen Hicks**

USA

**Oriane Hidalgo**

UK

**Éva Hideg**

Hungary

**Angelika Hilbeck**

Switzerland

**Henk Hilhorst**

Netherlands

**Ross Hill**

Australia

**Bradley Hillman**

USA

**Medrano Hipolito**

Spain

**Bertrand Hirel**

France

**Candice Hirsch**

USA

**Cory Hirsch**

USA

**Joseph Hirschberg**

Israel

**Ute Hoecker**

Germany

**Susanne Hoffmann-Benning**

USA

**Monica Hofte**

Belgium

**Annette Hohe**

Germany

**Yiguo Hong**

China

**Sharon Hook**

Australia

**Jim Horn**

USA

**Matthew Horton**

Austria

**David Horvath**

USA

**Li-Ching Hsieh**

Taiwan

**Jianping Hu**

USA

**Jinling Huang**

USA

**Zhenying Huang**

China

**Karen Hudson**

USA

**Alisa Huffaker**

USA

**Matthew Hufford**

USA

**Edgar Huitema**

UK

**Cristian Ibanez**

Chile

**Yoko Iijima**

Japan

**Tatsuya Ikeda**

Japan

**Richard Immink**

Netherlands

**Claudio Inostroza-Blancheteau**

Chile

**Rosy Isaias**

Brazil

**Nobuaki Ishida**

Japan

**Yasuhiro Ito**

Japan

**Norberto Iusem**

Argentina

**Rumen Ivanov**

Germany

**Hiroyoshi Iwata**

Japan

**Kunio Iwatsuki**

Japan

**Niranjani Iyer**

USA

**Takeshi Izawa**

Japan

**Jeanne Jacobs**

New Zealand

**Dan Jacobson**

South Africa

**Guru Jagadeeswaran**

USA

**Pradeep Jain**

India

**Malgorzata Jakubowicz**

Poland

**Georg Jander**

USA

**Robert Jansen**

USA

**Dennis Janz**

Germany

**Jose Jarillo**

Spain

**Paul Jarvis**

UK

**Amir Reza Jassbi**

Japan

**Eric Jenczewski**

UK

**Pablo Jenik**

USA

**Martin Jensen**

Denmark

**Soon-Chun Jeong**

Korea, South

**Holger Jeske**

Germany

**Heyuan Jiang**

China

**Ning Jiang**

USA

**Xingyu Jiang**

China

**Zhiqiang Jin**

China

**Biao Jin**

China

**Jian Jin**

China

**Hai-Chun Jing**

China

**Russell Jones**

USA

**Matthieu Henri Antoon Jozef Joosten**

Netherlands

**Angela Juhasz**

Hungary

**Je Hyeong Jung**

USA

**Geunhwa Jung**

USA

**Parijat Juvale**

USA

**Piotr Kachlicki**

Poland

**Pradeep Kachroo**

USA

**Joanna Kacprzyk**

Ireland

**Lei Kai**

Germany

**Vijaya Gopal Kakani**

USA

**Peter Kalo**

Hungary

**Sekhar Kambakam**

USA

**Radek Kana**

Czech Republic

**Angelos Kanellis**

Greece

**Byung-Ho Kang**

USA

**Hunseung Kang**

Korea, South

**Hong-Gu Kang**

USA

**Merijn Kant**

Netherlands

**Michael Kantar**

USA

**Kostya Kanyuka**

UK

**Rick Karban**

USA

**Surekha Katiyar-Agarwal**

India

**Kerstin Kaufmann**

Germany

**Jagreet Kaur**

India

**Nat Kav**

Canada

**Tsutomu Kawasaki**

Japan

**Kemal Kazan**

Australia

**Julia Kehr**

Spain

**Julia Kehr**

Germany

**Beat Keller**

Switzerland

**Felix Kessler**

Switzerland

**Zhang Kewei**

China

**Awais Khan**

Peru

**Aruna Kilaru**

USA

**Graham King**

Australia

**Edward Kirby**

USA

**Tatjana Kleine**

Germany

**Helmut Knüpffer**

Germany

**Takanori Kobayashi**

Japan

**Koichi Kobayashi**

Japan

**Michael Kobor**

Canada

**Andriy Kochevenko**

Germany

**Gábor Kocsy**

Hungary

**Ajay Kohli**

Philippines

**Bryan Kolaczkowski**

USA

**Frederic Kolb**

USA

**David Kopecky**

Czech Republic

**Helena Korpelainen**

Finland

**Jens Kossmann**

South Africa

**Simeon Kotchoni**

USA

**Stefanos Koundouras**

Greece

**Kanako Koyanagi**

Japan

**Elena Kramer**

USA

**Gabi Krczal**

Germany

**Sergey Krupenko**

USA

**Karl Kugler**

Germany

**Masahiko Kumagai**

Japan

**V. Dinesh Kumar**

India

**Sanjay Kumar**

India

**Shigeichi Kumazaki**

Japan

**Jochen Kumlehn**

Germany

**Carlos Labate**

Brazil

**Maite Lacuesta**

Spain

**Zhongxiong Lai**

China

**Zhibing Lai**

USA

**Thierry Langin**

France

**Maria Valeria Lara**

Argentina

**Debbie Laudencia-Chingcuanco**

USA

**Stefan Laurent**

Germany

**Virginie Lauvergeat**

France

**Thomas Laux**

Germany

**Erwan Le Deunff**

France

**Peter John Lea**

UK

**Tong Geon Lee**

USA

**Jong Kyu Lee**

Korea, South

**Suk-Ha Lee**

Korea, South

**Matti Leino**

Sweden

**Andrew Leitch**

UK

**Janne Lempe**

Germany

**Michael Lenhard**

Germany

**Bosheng Li**

USA

**Chenghao Li**

China

**Jiaru Li**

China

**Lei Li**

USA

**Huihui Li**

China

**Maoteng Li**

China

**Yuhua Li**

China

**Shutian Li**

Germany

**Song Li**

USA

**Hong-Ye Li**

China

**Ting Li**

USA

**Yan Li**

China

**Ying Li**

USA

**Yongfang Li**

USA

**Yongle Li**

Australia

**Guohua Liang**

China

**Marc Libault**

USA

**David Lightfoot**

USA

**Wilco Ligterink**

Netherlands

**Jun Lim**

Korea, South

**Pyung Ok Lim**

Korea, South

**Chentao Lin**

USA

**Jinxing Lin**

China

**Kui Lin**

China

**Yao-Cheng Lin**

Belgium

**Hong-Qing Ling**

China

**Alexander Lipka**

USA

**Jinjie Liu**

USA

**Jianquan Liu**

China

**Qingpo Liu**

China

**Shengyi Liu**

China

**Lai-Hua Liu**

China

**Wen-Zhe Liu**

China

**Renyi Liu**

USA

**Renyi Liu**

China

**Xigang Liu**

China

**Zhongchi Liu**

USA

**Alan Lloyd**

USA

**Clive Lo**

Hong Kong

**Maria Logacheva**

Russian Federation

**Susan Lolle**

Canada

**Jose Lopez-Bucio**

Mexico

**Rodolfo Lopez-Gomez**

Mexico

**Ann Loraine**

USA

**Aaron Lorenz**

USA

**Rafael Lozano**

Spain

**Hua Lu**

USA

**Yingmin Lu**

China

**Uwe Ludewig**

Germany

**Jutta Ludwig-Müller**

Germany

**Yan Luo**

China

**Qing-Hu Ma**

China

**Wujun Ma**

Australia

**Marco Maccaferri**

Italy

**Emma Mace**

Australia

**Milagros Machinandiarena**

Argentina

**David Mackey**

USA

**David Mackill**

USA

**Richard Colin Macknight**

New Zealand

**Takaki Maekawa**

Germany

**Massimo Maffei**

Italy

**Jurandir Magalhaes**

Brazil

**Ivan Maia**

Brazil

**Indu Maiti**

USA

**Mario Malagoli**

Italy

**Abul Kalam Mandal**

India

**Long Mao**

China

**Daunay Marie-Christine**

France

**Soma Marla**

India

**Cathie Martin**

UK

**Antonio Martín**

Spain

**Pedro Martinez-Gomez**

Spain

**José M Martinez-Zapater**

Spain

**Enrico Martinoia**

Switzerland

**Martin Mascher**

Germany

**Stefania Masci**

Italy

**Esten Mason**

USA

**Anna Maria Mastrangelo**

Italy

**Ulrike Mathesius**

Australia

**Andrea Matros**

Germany

**Fumio Matsuda**

Japan

**Hideaki Matsumoto**

Japan

**Roberto Mattioli**

Italy

**Tim Mauchline**

UK

**Brigitte Mauch-Mani**

Switzerland

**Ivan Maureira Butler**

Chile

**Klaus Mayer**

Germany

**Christian Mazars**

France

**Michael Mazourek**

USA

**Martin Robert Mcainsh**

UK

**Bruce Mcclure**

USA

**Karen Mcginnis**

USA

**J. Mitchell Mcgrath**

USA

**Leah Mchale**

USA

**Michael Mcmullen**

USA

**Joel Mcneal**

USA

**Kevin Mcphee**

USA

**Andy Meharg**

UK

**David Meinke**

USA

**Charles Melnyk**

UK

**Rainer Melzer**

Germany

**David Mendoza**

USA

**Yijun Meng**

China

**Zheng Meng**

China

**Marcelo Menossi**

Brazil

**Kristin Mercer**

USA

**Irute Meskiene**

Austria

**Jean-Pierre Metraux**

Switzerland

**Eli Meyer**

USA

**Rhonda Meyer**

Germany

**Blake Meyers**

USA

**Thomas Miedaner**

Germany

**Tony Millar**

Australia

**Tony Miller**

UK

**Joel Milner**

UK

**Ray Ming**

USA

**Mehdi Mirzaei**

Australia

**Richard Mithen**

UK

**Juliane Mittasch**

Germany

**Yutaka Miyazawa**

Japan

**Hans-Peter Mock**

Germany

**Wolfgang Moeder**

Canada

**Alejandra Moenne**

Chile

**Yvan Moënne-Loccoz**

France

**Volker Mohler**

Germany

**Ian Max Møller**

Denmark

**Tapan Mondal**

India

**Paula Maria Moolhuijzen**

Australia

**Richard Moore**

USA

**María De La Luz Mora**

Chile

**Raphael Morillon**

Spain

**Panagiotis Moschou**

Sweden

**Reiko Motohashi**

Japan

**Zhonglin Mou**

USA

**Katriina Mouhu**

Finland

**Iva Mozgova**

Sweden

**Wellington Muchero**

USA

**Gary Muehlbauer**

USA

**Douglas Muench**

Canada

**Sunil Mukherjee**

India

**Philip Mullineaux**

UK

**Sujatha Mulpuri**

India

**Stéphane Muños**

France

**Luis Mur**

UK

**Koji Murai**

Japan

**Koji Muramoto**

Japan

**Alex Murphy**

UK

**Denis Murphy**

UK

**Serge Muyldermans**

Belgium

**Sean Myles**

Canada

**Rachel Naegele**

USA

**Istvan Nagy**

Hungary

**Ken Naito**

Japan

**Kenta Nakai**

Japan

**Norihito Nakamichi**

Japan

**Kazuo Nakashima**

Japan

**Eiji Nambara**

Canada

**Simona Nardozza**

New Zealand

**Javier Narvaez-Vasquez**

USA

**Utpal Nath**

India

**Alireza Navabi**

Canada

**Suresh Neethirajan**

Canada

**Karlie Neilson**

Australia

**David Nelson**

USA

**Lev Nemchinov**

USA

**Madhav Nepal**

USA

**Carl Ng**

Ireland

**Henry Nguyen**

USA

**Dahlia Nielsen**

USA

**Tom Hamborg Nielsen**

Denmark

**Manuel Nieves-Cordones**

France

**Tomotaro Nishikawa**

Japan

**Takumi Nishiuchi**

Japan

**Ill-Sup Nou**

Korea, South

**Thomas Nussbaumer**

Austria

**Sergio J Ochatt**

France

**Francis Ogbonnaya**

Australia

**John Ohlrogge**

USA

**Akemi Ohmiya**

Japan

**Takashi Okada**

Australia

**Elina Oksanen**

Finland

**Kazutoshi Okuno**

Japan

**Richard Olmstead**

USA

**Matthew Olson**

USA

**Satoshi Oota**

Japan

**David Oppenheimer**

USA

**Bernardo Ordas**

Spain

**Jamie O'Rourke**

USA

**Cristina Ortega-Villasante**

Spain

**Anne Osbourn**

UK

**Eileen O'Toole**

USA

**Primoz Oven**

Slovenia

**Hakan Ozkan**

Turkey

**Natalia Pabon Mora**

Colombia

**Jordon Pace**

USA

**Chiara Pagliarani**

Italy

**Magda Pál**

Hungary

**Norman Pammenter**

Suriname

**Jianwei Pan**

China

**Sona Pandey**

USA

**Ralph Panstruga**

Germany

**Spiros Papakostas**

Finland

**Roger Parish**

Australia

**Gisele Passaia**

Brazil

**Maria Marta Pastina**

Brazil

**Victoria Pastor**

Switzerland

**Matthew Paul**

UK

**Katharina Pawlowski**

Sweden

**Tomasz Pawlowski**

Poland

**Wojtek Pawlowski**

USA

**Raja Payyavula**

USA

**Mario Enrico Pè**

Italy

**Scott Peck**

USA

**Xinwu Pei**

China

**Suprasanna Penna**

India

**Deepak Pental**

India

**Andy Pereira**

USA

**Silvia Pereyra**

Uruguay

**Begona Perez-Vich**

Sudan

**Serena Perilli**

Netherlands

**Dragan Perovic**

Germany

**Christoph Peterhansel**

Germany

**Sander Peters**

Netherlands

**Thomas Peterson**

USA

**Mario Pezzotti**

Italy

**Anh Pham**

USA

**Romain Philippe**

France

**Pietro Piffanelli**

Italy

**Sara Pinosio**

Italy

**J. Chris Pires**

USA

**Frederic Pitre**

Canada

**Georg Pohnert**

Germany

**Benoit Poinssot**

France

**Danijela Poljuha**

Croatia

**Andrea Polle**

Germany

**Mikhail M. Pooggin**

Switzerland

**Zoe Popper**

Ireland

**David Pot**

USA

**Sreedhara Ashok Prabhu**

India

**Manoj Prasad**

India

**Jill Preston**

USA

**Holger Puchta**

Germany

**Stanislawa Pukacka**

Poland

**Jo Putterill**

New Zealand

**Xiaoquan Qi**

China

**Xiaoquan Qi**

UK

**Feng Qin**

China

**Wenping Qiu**

USA

**Le Qing Qu**

China

**Ronaldo Quaggio**

Brazil

**Silvia Quaggiotti**

Italy

**Marie-Christine Quillet**

France

**Sathishkumar Ramalingam**

India

**Kanniah Rajasekaran**

USA

**Randeep Rakwal**

Japan

**Harsh Raman**

Australia

**Jean-Francois Rami**

France

**Xie Rangjin**

China

**R. George Ratcliffe**

UK

**Milind Ratnaparkhe**

India

**Catherine Ravel**

France

**Bianca Reeksting**

South Africa

**Tony Remans**

Belgium

**Maria Remis**

Argentina

**Hugues Renault**

France

**Ralf Reski**

Germany

**Manuel Rey**

Spain

**Jose Luis Reyes**

Mexico

**Arantza Rico**

UK

**Christoph Ringli**

Switzerland

**Rosa M Rivero**

Spain

**Dae-Kyun Ro**

Canada

**Helene Robert**

Czech Republic

**Irma Roberts**

Argentina

**Monica Rodriguez**

Italy

**Carlos Marcelino Rodriguez Lopez**

Australia

**Adrienne Roeder**

USA

**William John Rogers**

Argentina

**Charles Romieu**

France

**Ann Christin Rönnberg-Wästljung**

Sweden

**William Rooney**

USA

**Luis Mauro Rosa**

Brazil

**Jocelyn Rose**

USA

**Ray J. Rose**

Australia

**Rebecca Roston**

USA

**Mathieu Rouard**

France

**Steve Rounsley**

USA

**Matthew Rouse**

USA

**Heather Rowe**

Canada

**Swarup Roy Choudhury**

USA

**Wilfried Rozhon**

Austria

**Markus Ruhsam**

UK

**Daniel Runcie**

USA

**John Runions**

UK

**Mark Running**

USA

**Bob Rutledge**

Canada

**Peter Ryan**

Australia

**Francois Sabot**

France

**Melanie Sacco**

USA

**Gavin Sacks**

USA

**Ari Sadanandom**

UK

**Kari Saikkonen**

Finland

**Katsumi Sakata**

Japan

**Keyan Salzman**

USA

**Deborah Samac**

USA

**Jozef Samaj**

Czech Republic

**Robert Sammons**

USA

**Marcus Samuel**

Canada

**Gerardo Sánchez**

Argentina

**Paloma Sanchez-Bel**

Spain

**Devinder Sandhu**

USA

**Mirella Sari Gorla**

Italy

**Tomasz Sarnowski**

Poland

**Hidenori Sassa**

Japan

**Kazuhiro Sato**

Japan

**Arnould Savoure**

France

**Samir Sawant**

India

**Rachit Saxena**

India

**Henk Schat**

Netherlands

**Günther Scherer**

Germany

**John Schiefelbein**

USA

**Florian P Schiestl**

Switzerland

**Jos Schippers**

Germany

**Philipp Schlüter**

Switzerland

**Gerald Schoenknecht**

USA

**Monika Schreiner**

Germany

**Gunda Schulte Auf'M Erley**

Germany

**Wilfried Schwab**

Germany

**Jorg Schwender**

USA

**Steven Scofield**

USA

**Marek Sebela**

Czech Republic

**Ronald Sederoff**

USA

**Armand Seguin**

Canada

**Verena Seidl-Seiboth**

Austria

**Georg Seifert**

Austria

**Yasuyo Sekiyama**

Japan

**David Selinger**

USA

**Jennifer Selinski**

Germany

**Marc-André Selosse**

France

**Champa Sengupta-Gopalan**

USA

**Muthappa Senthil-Kumar**

India

**Shigemi Seo**

Japan

**Kjell Sergeant**

Luxembourg

**Andrew Severin**

USA

**Jyoti Shah**

USA

**Zaigham Shahzad**

France

**Libo Shan**

USA

**Eilon Shani**

Israel

**Thomas Sharkey**

USA

**Peter Sharp**

Australia

**Yuri Shavrukov**

Australia

**Cheng Shihua**

China

**Toshiharu Shikanai**

Japan

**Kaori Shiojiri**

Japan

**Katsuhiro Shiratake**

Japan

**Qing Yan Shu**

China

**Bin Shuai**

USA

**Jeffry Shultz**

USA

**Cao Shuqing**

China

**Richard Sicher**

USA

**Imran Siddiqi**

India

**Lyudmila Sidorenko**

UK

**Daniela Sieh**

Germany

**Herman Silva**

Chile

**Thierry Simonneau**

France

**Anil Singh**

India

**Dharmendra Singh**

USA

**Mohan Singh**

Australia

**Alok Krishna Sinha**

India

**Aleksandra Skirycz**

Brazil

**Leif Skot**

UK

**Mark Smith**

Canada

**Kevin Smith**

USA

**Petr Smykal**

Czech Republic

**David Smyth**

Australia

**Kimberley Snowden**

New Zealand

**Michal Sochor**

Czech Republic

**Moon-Soo Soh**

Korea, South

**Juan Solomon**

USA

**Rentao Song**

China

**Gabriella Sonnante**

Italy

**Jose Miguel Soriano Soriano**

Italy

**Nathan Springer**

USA

**Sheshshayee Sreeman**

India

**Nese Sreenivasulu**

Germany

**Rakesh Srivastava**

India

**Margaret Staton**

USA

**Katherine Steele**

UK

**Nils Stein**

Germany

**Trevor Stevenson**

Australia

**C Neal Stewart**

USA

**Annick Stintzi**

Germany

**Maciej Stobiecki**

Poland

**Lucia Strader**

USA

**Koichi Sugimoto**

Japan

**Genlou Sun**

Canada

**Qi Sun**

USA

**Wenxian Sun**

China

**Sridevi Sureshkumar**

Australia

**Fedora Sutton**

USA

**Stephanie Swarbreck**

UK

**Kelly Swarts**

USA

**Laszlo Szabados**

Hungary

**Jedrzej Szymanski**

Israel

**Alice Tadiello**

Italy

**Helen Tai**

Canada

**Junji Takabayashi**

Japan

**Taku Takahashi**

Japan

**Toshiyuki Takai**

Japan

**Naoki Takata**

Japan

**Shigeo Takumi**

Japan

**Tsuyoshi Tanaka**

Japan

**Yoshikazu Tanaka**

Japan

**Jihua Tang**

China

**Georgia Tanou**

Greece

**Celine Tasset**

Australia

**Massimiliano Tattini**

Italy

**Noemi Tel-Zur**

Israel

**Francisco Tenllado**

Spain

**Jason Terpolilli**

Australia

**Valeria Terzi**

Italy

**Pilar S. Testillano**

Spain

**Anja Thalhammer**

Germany

**Nguyen Phuong Thao**

Viet Nam

**Guenter Theissen**

Germany

**Gerhard Thiel**

Germany

**Marco Thines**

Germany

**Sébastien Thomine**

France

**Michael Thomson**

Philippines

**Corinna Thurow**

Germany

**Li Tian**

USA

**Denise Tieman**

USA

**Peter Tiffin**

USA

**Mikko Tikkanen**

Finland

**Michael Timko**

USA

**Louis Tisa**

USA

**Alain Tissier**

Germany

**Takayuki Tohge**

Germany

**Seiichi Toki**

Japan

**J Tokuhisa**

USA

**Alessandro Tondelli**

Italy

**Kinya Toriyama**

Japan

**Paola Tosi**

UK

**Emily Tozzi**

USA

**Lam-Son Tran**

Japan

**Leena Tripathi**

Kenya

**Vineeta Tripathi**

India

**Tokuji Tsuchiya**

China

**Lilu Tu**

China

**Matthew Tucker**

Australia

**Paul Tudzynski**

Germany

**Rickie Turley**

USA

**Ludmila Tyler**

USA

**Joshua Udall**

USA

**Saneyoshi Ueno**

Japan

**Kristian Ullrich**

Germany

**Roman Ulm**

Switzerland

**Peter Ulvskov**

Denmark

**Mikihisa Umehara**

Japan

**Shimpei Uraguchi**

Japan

**Bjorn Usadel**

Germany

**Roland Valcke**

Belgium

**Esther Van Der Knaap**

USA

**C. Gerard Van Der Linden**

Netherlands

**Leon Van Eck**

USA

**Joost Van Heerwaarden**

Netherlands

**Sjaak Van Heusden**

Netherlands

**Eibertus Nicolaas Van Loo**

Netherlands

**Gerben Van Ooijen**

UK

**Leo Van Overbeek**

Netherlands

**Klaas Van Wijk**

USA

**Michiel Vandenbussche**

France

**Kranthi Varala**

USA

**Felicity Vear**

France

**Leonardo Velasco**

Sri Lanka

**Ignazio Verde**

Italy

**Paul Verslues**

Taiwan

**Filipe Victoria**

Brazil

**Sabina Vidal**

Uruguay

**Maria Lucia Carneiro Vieira**

Brazil

**Martin Vila Aiub**

Argentina

**Patrick Vincourt**

France

**Pavel Vítámvás**

Czech Republic

**Paola Vittorioso**

Italy

**Melane Alethea Vivier**

South Africa

**Konstantinos Vlachonasios**

Greece

**Thomas Vogt**

Germany

**Hugo Volkaert**

Thailand

**Albrecht Von Arnim**

USA

**Mandy Walker**

Australia

**John Walker**

USA

**Ian Wallace**

USA

**John Walsh**

UK

**Michael H. Walter**

Germany

**Yongfang Wan**

UK

**Aiming Wang**

Canada

**Yu Fu Wang**

China

**Daowen Wang**

China

**Li Wang**

China

**Lijun Wang**

China

**Ming-Bo Wang**

Australia

**Mei Wang**

China

**Chung-Ju Wang**

Taiwan

**Junqi Wang**

China

**Baichen Wang**

China

**Guixi Wang**

China

**Hao Wang**

Hong Kong

**Jianying Wang**

USA

**Shucai Wang**

China

**Xingjun Wang**

China

**Xuelu Wang**

China

**Yi Wang**

China

**Ying Wang**

China

**Hao Wang**

USA

**Brian Waters**

USA

**Jill Wegrzyn**

USA

**Dongmei Wei**

China

**Shu Wei**

China

**Ralf Welsch**

Germany

**Susann Wicke**

Germany

**Olivia Wilkins**

USA

**Eva-Maria Willing**

Germany

**Iain Wilson**

Australia

**Karl Wilson**

USA

**Zoe Wilson**

UK

**Kamil Witek**

UK

**Claus-Peter Witte**

Germany

**Sebastian Wolf**

Germany

**Marnin Wolfe**

USA

**Anne-Marie Wolters**

Netherlands

**Darren Wong**

Canada

**Feibo Wu**

China

**Xiaoming Wu**

China

**Yisheng Wu**

USA

**Nelson Wulff**

Brazil

**Bernhard Wurzinger**

Austria

**Gui-Xian Xia**

China

**Deyu Xie**

USA

**Zhixin Xie**

USA

**Yongzhong Xing**

China

**Kevin Xiong**

USA

**Changjie Xu**

China

**Chunming Xu**

China

**Huini Xu**

China

**Mingliang Xu**

China

**Lin Xu**

China

**Liangjiao Xue**

USA

**Yukinori Yabuta**

Japan

**Igor Yakovlev**

Norway

**Toshiya Yamamoto**

Japan

**Jun Yan**

USA

**Huimin Yang**

China

**Ming Yang**

USA

**Qing Yang**

China

**Tae-Jin Yang**

Korea, South

**Yi Yang**

China

**Guangxiao Yang**

China

**Yinong Yang**

USA

**Zhong-Nan Yang**

China

**Yu Yanli**

China

**Quanhong Yao**

China

**Yingyin Yao**

China

**Hideshi Yasui**

Japan

**Huaxun Ye**

USA

**Zhuoliang Ye**

USA

**Kuo-Chen Yeh**

Taiwan

**Bin Yi**

China

**Yanhai Yin**

USA

**Cheol-Min Yoo**

USA

**Mi-Jeong Yoo**

USA

**Keisuke Yoshida**

Japan

**Kumi Yoshida**

Japan

**Keiko Yoshioka**

Canada

**Bin Yu**

USA

**Jianming Yu**

USA

**Tien-Shin Yu**

Taiwan

**Xiaolin Yu**

China

**Jingjuan Yu**

China

**Olga Yurchenko**

USA

**Lorenzo Zacarias**

Sri Lanka

**Zbynek Zdrahal**

Czech Republic

**Dali Zeng**

China

**Chunquan Zhang**

USA

**Deqiang Zhang**

China

**Dong Zhang**

USA

**Jingjuan Zhang**

Australia

**Jian Zhang**

Canada

**Jinfa Zhang**

USA

**Jin-Song Zhang**

China

**Lizhi Zhang**

USA

**Guoquan Zhang**

China

**Liang Zhang**

China

**Xian-Chun Zhang**

China

**Yansheng Zhang**

China

**Zhanyuan Zhang**

USA

**Zhiming Zhang**

China

**Huixian Zhao**

China

**Meixia Zhao**

USA

**Hongbo Zhao**

China

**Chengchao Zheng**

China

**Shao Jian Zheng**

China

**Wenguang Zheng**

USA

**Jun Zheng**

China

**Adele Zhou**

USA

**Meixue Zhou**

Australia

**Weijun Zhou**

China

**Yongming Zhou**

China

**Jianhua Zhu**

USA

**Changfu Zhu**

Spain

**Danmeng Zhu**

China

**Jörg Ziegler**

Germany

**Agnieszka Zienkiewicz**

USA

**Piotr Ziolkowski**

Poland

**Kaijing Zuo**

China

**Matias Zurbriggen**

Germany

**Eva Zyprian**

Germany

